# Taking patient involvement seriously: a critical ethical analysis of participatory approaches in data-intensive medical research

**DOI:** 10.1186/s12911-019-0799-7

**Published:** 2019-04-25

**Authors:** Katharina Beier, Mark Schweda, Silke Schicktanz

**Affiliations:** 1 Department of Medical Ethics and History of Medicine, Georg-August-University Göttingen, University Medical Center, Humboldtallee 36, 37073 Göttingen, Germany; 2 Department of Health Services Research, Carl von Ossietzky University of Oldenburg, School for Medicine and Health Sciences, Ammerländer Heerstr. 114-118, 26111 Oldenburg, Germany

**Keywords:** Patient participation, Empowerment, Participatory health research, Research ethics, Bioethics, Big data

## Abstract

**Background:**

Data-intensive research in medicine and healthcare such as health-related big data research (HBDR) implies that data from clinical routine, research and patient-reported data, but also non-medical social or demographic data, are aggregated and linked in order to optimize biomedical research. In this context, notions of patient participation and involvement are frequently invoked to legitimize this kind of research and improve its governance. The aim of this debate paper is to critically examine the specific use and ethical role of participatory concepts in the context of HBDR and data-intensive research in medicine and healthcare.

**Discussion:**

We introduce basic conceptual distinctions for the understanding of participation by looking at relevant fields of application in politics, bioethics and medical research. Against this backdrop, we identify three paradigmatic participatory roles that patients/subjects are assigned within the field of HBDR: participants as providers of biomaterials and data, participants as administrators of their own research participation and participants as (co-)principal investigators. We further illustrate these roles by exemplary data-intensive research-initiatives. Our analysis of these initiatives and their respective participatory promises reveals specific ethical and practical shortcomings and challenges. Central problems affecting, amongst others, ethical and methodological research standards, as well as public trust in research, result from the negligence of essential political-ethical dimensions of genuine participation.

**Conclusions:**

Based on the conceptual distinctions introduced, we formulate basic criteria for justified appeals to participatory approaches in HBDR and data-intensive research in medicine and healthcare in order to overcome these shortcomings. As we suggest, this is not only a matter of conceptual clarity, but a crucial requirement for maintaining ethical standards and trust in HBDR and related medical research.

## Background

In 2013, the so-called ‘*care.data*’ scheme was introduced in the UK. It aimed at collecting the medical information of all patients treated within the National Health Service (NHS) in a central database that was open to researchers and others, unless patients explicitly opted out. This initiative was promoted as supporting patients’ choices, and visions of enhanced patient empowerment were invoked by the media. The Guardian, for example, ran the headline: “Open access to data can break down barriers and empower patients” [1]. After its introduction, however, the *care.data* scheme was heavily criticized by general practitioners and citizens. A website set up by the NHS to learn about citizens’ concerns revealed major criticism targeted at the lack of transparency, as well as insufficient prior consultation of and communication with the public [2]. As a consequence, and after the release of the Caldicott-report by the National Data Guardian for Health and Care, *care.data* was shut down in 2016. The report called for an intensified dialogue with the public in order to restore their trust [3].

This example illustrates the current boom of appeals to 'public engagement' in the context of data-intensive research in medicine and healthcare [4]. At the same time, however, it shows how failure to live up to claims to public engagement can threaten the success of such research initiatives. In general, the respective kind of research is often discussed under the label of 'big data'. Although the concept of ‘big data’ is still vague and contested [5], it usually refers to large data volumes characterized by a high velocity, complexity and variety, whose collection, storage, management and analysis demand new information technological solutions [6]. In the medical context, this implies that data from clinical routine, research and patient-reported data, but also non-medical, even unstructured, social or demographic data, are aggregated and linked in order to optimize biomedical research. This development of health-related big data research (HBDR) is accompanied by high hopes for more personalized treatments, as the merging of large data sets from different sources is expected to facilitate disease prediction as well as treatment decisions through the stratification of patients according to specific disease subgroups. In light of this vision, the potential for fundamentally improving healthcare is often ascribed to HBDR [7, 8]. Although a return to small data can be observed in some contexts [9], many health policy initiatives still emphasize big data. To understand complex diseases, as well as multifactorial interactions between different potentially disease-related variables, large data volumes must be analysed. This is particularly relevant for rare diseases or diseases with unknown causes. In these contexts, data-driven approaches are deemed more promising than hypothesis-driven research strategies. Since it is hard to tell which data will be useful in future research, the generation of large data sets is often seen as advisable in this context. In Germany, for example, the federally funded *Medical Informatics Initiative* aims to establish an infrastructure for the integration of a broad range of clinical and research data [10].

In this paper, we choose the umbrella term ‘data-intensive research’ to cover the variety of approaches to the digitalized collection and analysis of larger sets of data, including but not limited to ‘big data’ in particular. The collection and analysis of such large data sets gives rise to a variety of ethical, legal and social concerns. In the European Data Protection Directive, medical data, due to their sensitivity, form a separate category that requires stricter regulation. Conceptualizing patient information and informed consent to ensure data sovereignty is particularly challenging when data from different sources are consolidated for future research purposes that cannot be specified in advance [11]. Although the Directive’s requirements are less strict for scientific research regarding the specification of using personal data (recital 33), this does not provide a carte blanche for any research use of personal data. For example, if medical data are collected at a large scale, it cannot be taken for granted that the data subject’s consent automatically covers all conceivable future research purposes. Moreover, while the data subject’s consent is seen as dispensable if the research in question concerns a significant public interest (recital 156), this may be more difficult to argue in the case of data-intensive research approaches that pursue open-ended purposes. While social stigmatization and discrimination can always result if medical data fall into wrong hands, the large-scale analysis of data from different sources increases the amount of sensitive information, e.g., by facilitating more fine-grained stratification. Furthermore, regulations on data protection, e.g., concerning anonymization, aim to protect individuals against problematic usage of their data. However, there is increasing awareness that traditional measures may no longer be sufficient as re-identification of individuals is getting easier the more data are available [12]. In this regard, medical research with large data sets inevitably entails personal data. Thus, data-intensive research approaches exacerbate the general risk of re-identification. Moreover, control over data use may no longer be attainable at the individual level, which raises not only issues regarding individual rights to data access, but also regarding participant withdrawal and data erasure [8, 13]. Since there are numerous ethical issues which cannot be resolved by ethical or legal experts alone, notions of patient participation and involvement (PPI)^1^ are frequently invoked in order to legitimize data-intensive research in medicine in general and HBDR in particular and improve its ethical and legal governance. This also implies promises of a new role for subjects: being approached as partners in research, or citizen researchers [14]. However, the meaning and practical implications of PPI in HBDR are far from clear. Even beyond this specific field, participation has been characterized as a vague concept [15]. The plurality of participatory terms used in the context of biomedical research exemplifies this confusion. Although there are certainly semantic overlaps between terms such as ‘patient involvement’, ‘patient engagement’, ‘patient participation’[4], ‘citizen science’ [16], ‘participant-centric initiatives’ [14], ‘patient-led research’ [17] or ‘participatory health research’ [18], the role these concepts assign to participants in research is neither clear nor homogeneous. From an ethical point of view, claims for participation show different levels of ambition and sophistication.

Against this backdrop, our paper focuses on the significance of ‘participation’ in HBDR and similar data-intensive research in medicine and healthcare. Discussing the whole range of other ethically relevant issues related to this kind of research is beyond the scope of the paper and cannot be realized in a systematic manner within the limited space. The central objective is to critically analyse the specific use and ethical role of participatory concepts in data-intensive research initiatives and discourses in medicine and healthcare. Admittedly, ‘participation’ has become an important topic in medical research in general (see below). Accordingly, many of the points addressed in the subsequent analyses may not be unique or exclusive to data-intensive research but also apply to other fields of medical science. Still, the current boom of participatory claims in the context of ‘big data’ and ‘digitalization’ in medicine and healthcare appears significant and hence deserves closer attention. This is all the more relevant as the range of involved disciplines is expanding far beyond medicine, life sciences and public health, by also including medical informatics, engineering, mathematics, and computer science. Our analysis of the specific use and ethical role of participatory concepts proceeds as follows: In the background section, we first introduce basic conceptual considerations regarding the *normative understanding of participation*. For this purpose, we refer to discussions in political theory and bioethics and reconstruct how they have impacted and shaped the meaning of participation in medical research. In doing so, we identify *three paradigmatic types of practical conditions* that constitute and define participation: (a) consent to individual provision of data or biomaterial, (b) consultation regarding research and health policy aims and (c) cooperation in decision-making processes or even involvment of participants as co-researchers. In the analysis and discussion section, we then consider exemplary research initiatives for each of these three types and *critically analyse specific ethical and practical challenges that arise from the accompanying participatory claims*. In the outlook section, we finally formulate *five ethical recommendations* for increasing the ethical legitimacy, trustworthiness and feasibility of participatory promises in the context of HBDR and similar data-intensive research in medicine and healthcare.

### History and theoretical framework of participation

The term '*participation'* originally comes from political discourse but was later adopted by bioethics and medical research [19]. In the following, we will develop a deeper understanding of the respective meaning and implications of participation in these three spheres. This will serve as a normative matrix for our subsequent analysis and evaluation of participatory promises in the field of data-intensive medical research such as HBDR.

### Political discourse

Criticisms regarding the effectiveness and legitimacy of Western representative democracies have fuelled calls for an intensified and more direct involvement of citizens in political matters since the 1960s/70s. In this context, participation was not only invoked as a means to improve the results of political decision-making, it was also assigned with the intrinsic value of realizing the ideal of democratic citizenship [20]. While public or citizen participation can take on various practical forms, there is a broad consensus that it needs to involve some transfer of power [21], so that those who are affected by a decision *have an input* into the respective decision-making process [22]. Underlying this is the original political ideal of autonomy in the sense of participation in collective self-government. Thus, political participation is not to be equated with mere communication strategies (e.g., the state informs citizens about political strategies or aims), but must grant citizens some real influence which is often understood as empowerment [23].

### Bioethical discourse

Participatory notions have also been influential in the field of bioethics. From the early 1970s onwards, bioethics can be characterized as an academic and political discourse dealing with ethical and social issues raised by medicine and the life sciences [24]. Since then, participatory notions have gained prominence in this field due to several factors. First, bioethics explicitly framed medical and biotechnological developments as a matter of general public concern. In this context, participation was suggested to complement the perspective of experts (e.g., doctors or bioethicists on review boards and in politics) and acknowledge the fact that lay persons are also capable of ethical reflection. This promoted a participatory turn in bioethics [25]. Besides this politically oriented motivation, there are also further ethical reasons for the inclusion of lay perspectives and lay moralities. In particular, if bioethical issues are only addressed by experts, other ethically relevant attitudes, concerns and conflicts may be neglected. For example, patients suffering from a disease may already find themselves in a vulnerable/disadvantaged position that can be exacerbated by paternalistic attitudes on the part of doctors or researchers. The involvement of patients can counteract this tendency by giving them greater control over decisions and actions affecting their health [26]. In the context of current empirically informed bioethics, this is frequently achieved by socio-empirical research exploring lay persons’ moral views and attitudes regarding ethical issues. On a political level, there are also participatory formats such as citizen or consensus conferences, where a randomly selected set of lay persons, more or less representative of the diversity of society, develop ethical recommendations on controversial biotechnological or biomedical developments [27].

### Medical research

Reference to participation is also increasingly made in the field of medical research itself [28]. This includes a wide scope ranging from community medicine, health services and public health studies to biomedical research. Apart from the involvement of patients in the development of clinical practice guidelines [29], patients can also be included in the research process as such. A precedent of such an inclusion was the promotion of early AIDS-research by grassroots activists [30]. Participatory research in medicine has been classified according to different models [31–33]. Although these models are of different theoretical origin and conceptual granularity, three overarching levels of patient participation can be identified:

- *Consent to research/treatment.* While this may appear as a rather obvious practice, it is important to note that, from a historical perspective, obtaining patients’/subjects’ consent has not always been self-evident [34]. The fact that patients themselves can decide whether they want to participate in a study grants them at least some influence on an individual level.
- *Consultation for setting the research agenda*, e.g., by defining general aims and limits of research [25, 35]. Notably, this can also be realized by the politically oriented participation of representatives of patient organizations or of groups of civil society in ethics boards, councils, or polls.
- *Involvement as co-researchers in definition of aims, design and implementation of research*.^2^ In particular, conceptions of participatory research in health or social sciences are garnering increasing resonance.^3^ In this context, lay persons whose life is affected by the research in question are co-determining the aims of research and may also act as co-producers of scientific knowledge by getting involved in all phases of the research process [36, 37]^4^.

Overall, appeals to participation in politics, bioethics and medical research cover a broad spectrum of activities. In all spheres, participation is linked to the idea of giving lay persons some power to influence opinion formation and decision-making where otherwise professionals (e.g., politicians, doctors, researchers) dominate. However, significant differences exist regarding the participatory dimension and degree of influence.

## Discussion: participation in data-intensive medical research

Although appeals to engagement and participation are frequent in the context of HBDR and other kinds of data-intensive research in medicine and healthcare, only few analyses are concerned with their normative foundations and ethical applications [38]. We therefore want to analyse the role of participatory claims in pertinent research initiatives and discourses in more depth. Against the background of the aforementioned three levels of participation – consent, consultation and cooperation – we highlight and discuss the three specific roles for participants for selected examples: A) participants as providers of data and biomaterials, B) participants as administrators of their own research participation, and C) participants as (co-) principal investigators. The following aim is to critically discuss the respective strengths and weaknesses of the underlying participatory roles in these examples from an ethical point of view.

### Individual consent: participants as providers of biomaterials and data

In order to improve the understanding of diseases and to develop future therapies, the availability of large data sets from a broad range of patients and healthy individuals is an important prerequisite. Increased efforts to enroll patients in data-intensive research may also come with new duties for them. For example, in support of a learning healthcare system, some ethicists argue for an obligation of patients to participate in activities that are likely to improve the quality of healthcare. They list activities such as participation in health registries, in reviews of de-identified medical records, and interviews focusing on the experiences and needs of patients [39].

The framing of data provision as an instance of participation is most frequent in the context of IT-based research technologies. That patients themselves can collect health-related data, for example, by using wearables, sensors or health-apps [40], or by simply filling out a questionnaire on a tablet computer [41], is often presented as a form of patient participation, citizen science and/or empowerment. *Apple* holds an own business segment for research that is solicited, amongst others, by the slogan “Empowering medical researchers, doctors, and you” [42]. Following this line of reasoning, it has been shown that the authors of the *Asthma Mobile Health* study, which is based on *Apple’s ResearchKit*, understand their project as an instance of citizen science, claiming that it “represents the coming together of academia and industry to benefit from the ubiquity of smartphones and harness the power of citizen-science to modernize the clinical research process” [43]. As another example, the diabetes network *TuAnalyze*, which allows patients not only to track their blood glucose levels, but also to share these data with researchers, is frequently referred to as an instance of citizen science [44].

Given that the collection of data has long been the unquestioned domain of researchers, the extension of data collection practices to patients themselves carries, at least superficially, a democratic and empowering potential: Rather than leaving data collection to the monopoly of medical professionals, patients themselves acquire this competence with the help of new technical devices. However, the use of smartphones or other electronic devices does not necessarily imply a qualitative difference for the involvement of patients in research. Rather than granting them a more active role, it primarily renders recruitment more convenient and effective *for researchers*. In fact, patients’ activity is largely restricted to the completion of surveys and the entrance of data, often on a daily basis. 5 In this sense, participatory rhetoric often simply embellishes a practical and cost-efficient outsourcing of research work [18].

In such a case, the power of citizen science is not promoted on a political level, but rather in the sense of ‘statistical power’. This becomes even more striking since participation often appears synonymous with ‘making a personal contribution’ to research. For example, although Weitzman and colleagues discuss *TuAnalyze* as an instance of citizen science, they describe it at the same time as “a model for engaging a distributed population of observers in research activities through reporting, sharing and contributing labour and computing time” [44]. This description is informative as it reflects the idea that patients are contributing to, but hardly influencing research.^6^ This corresponds to what Chung and Lounsburry define as traditional or compliant research [30].

From an ethical perspective, however, it is problematic to gloss over this kind of patient work [18] by using participatory and emancipatory language. This is not only a matter of conceptual honesty but also of immediate practical importance and moral integrity. If recruitment for research builds on participatory promises that are not fulfilled or even strategically used to exploit participants’ good will and engagement, this may significantly impair public trust in science as the case study of *data.care* indicates. Even though large data sets are essential in this kind of research, participatory language must not degenerate into a merely strategic tool to enrol research participants [14]. This way, participation would be robbed of its far more ambitious ethical implications. In the light of patients’ usually high willingness to contribute biomaterials and data to research [45, 46], the use of participatory notions to motivate enrolment in data-intensive medical research such as HBDR can be questioned. Overall, the ethical challenge here is to ensure valid consent models preventing the use of data or biomaterial without the knowledge or consent of participants or even against their explicit will [47]. In certain research contexts, sound and reliable data protection regimes and transparent information may actually be more important to patients than prospects for getting actively involved in data collection.

### Consultation: participants as administrators of their own research participation

Public consultation and deliberation about the general aims of research form an integral aspect in different conceptualizations of participation in medical research. At first sight, this vision is also present in the context of HBDR and other kinds of data-intensive research in medicine and healthcare. Using 'patient-centric initiatives' as an umbrella term, Kaye and colleagues subsume a broad range of tools, programs and projects that empower participants to engage in the research process using IT. As one of four key functions, they identify dynamic negotiation which is said to facilitate a continuous discourse and negotiation between researchers and participants and allows participants to manage their preferences about who is authorized to access and use their data [14].

However, a closer look at current initiatives reveals a shift of emphasis in the understanding of consultation. For example, the US-based platform *Private Access* provides an online tool for the management of personal health information. The website states that “Private Access empowers participants to share data with whomever they wish”. In practice, this means that participants can manage, amongst others, their preferences regarding consent procedures, data access, re-contact, withdrawal or feedback on an ongoing basis. Based on this information, “patients can be matched to appropriate clinical trials through the click of a mouse” [48]. It is striking, however, that neither consultation nor negotiation about research as such take place in this context. Eventually, the ‘click of a mouse’ constitutes an individual, largely administrative act rather than a mutual, dialogic interaction. Even though patients can manage the mode and the extent of their contribution via online tools, this does not necessarily imply that the relationship and exchange with researchers becomes closer, nor that they are given an actual say over the direction of research.

The direct-to-consumer genetic testing company *23andMe* provides another illustrative example in this regard. The company not only offers personalized genetic information to registered users, but also builds its own research database. *23andMe* raises a broad range of ethical questions [49], e.g., regarding participants’ informed and voluntary consent and their awareness of the use of their data for specific research purposes. In our context, the company’s participatory rhetoric is of particular relevance. *23andMe* solicits its offer by slogans like “Becoming part of something bigger: Our genetic research gives everyday people the opportunity to make a difference by participating in a new kind of research — online, from anywhere” [50]. Participants who provide their saliva and related data are approached as individuals who can make – as members of a collective – an impact on research by allowing their data to be used. However, this bottom-up approach is thwarted by the fact that behind the interactive façade of *23andMe*, a commercial interest looms large [33]. According to this rationale, people’s voice in research is only of interest if it is profitable. Goldberg points out that on the internet there is no ‘debating and deliberating’ that is not also ‘buying and selling’ [51]. Although participants in *23andMe* are regularly informed about ongoing research projects, they are still more adequately described as valuable but largely passive data sources rather than active participants who are given a voice over the direction and accomplishment of research.

This raises the question why the terminology of citizen science, participation or empowerment has become so influential in online communication tools in this context. A potential explanation might be the impending loss of control over the use of data [14, 52]. On this premise, online platforms – insofar as they allow patients to maintain control over their data – may be ascribed an empowering effect. However, it is important to note that the kind of power that is granted to participants here is still an impoverished one compared to the genuine ethical-political understanding of participation introduced above. In particular, it is an erroneous assumption to expect that genuine mutual consultation and deliberation will emerge as a natural by-product of online-tools. In fact, genuine consultation and deliberation in research might be better achievable by including, for example, patient representatives in the governing bodies of research-initiatives. Currently, this more indirect mode of participation is hardly an issue in the respective debates. However, since consultation about the aims of research at the individual level may neither be effective nor ethically always required, it seems worthwhile to harness the potential of representation for future HBDR and other kinds of data-intensive research in medicine and healthcare.

### Co-operation: participants as (co-) principal investigators

The inclusion of patients and subjects as co-decision-makers and co-researchers can be seen as the most straightforward realization of citizen science. From the perspective of the proponents of participatory health research, only the involvement of lay participants throughout all phases of the research process can legitimately be classified as participation [36]. However, if we apply this condition to the field of data-intensive research in medicine and healthcare, only a small segment of current initiatives can be rightfully assessed as actually being participatory.

The US-based private company *Patients.Like.Me* (PLM), for example, does not only ask for patients’ data, but allows them to compile own research cohorts. A much-cited example of this form of engagement is the so-called Lithium study. In reaction to a study that suggested a decelerating effect of Lithium on the progression of Amyotrophic Lateral Sclerosis (ALS) based on a cohort of 28 patients, a cohort of 348 ALS patients who organized themselves via PLM decided to take Lithium and was able to rebut the result [53].

From an ethical perspective, this example appears closest to what was described as empowering co-investigation [31]. Here, the original political-ethical spirit of participation is retained: the transfer of power. An even more far-reaching, though rarely practiced approach to establishing a power balance between researchers and patients would also require equal access to funding and relevant sources of knowledge production (e.g., scientific journals). However, from a research-ethical and methodological perspective, granting participants the role of co-decision-makers or co-researchers does not come without challenges. Most strikingly, if the use of pharmaceuticals, for example, is tested in self-initiated trials beyond the safeguards of traditional research ethics, e.g., ethical review, assurance of free and informed consent, prior assessment of the benefits-risk-ratio etc., this can imply serious risks to participants. Particularly terminally ill patients may be willing to accept massive risk exposure.

Moreover, the scientific rigor of the results may be compromised for several reasons. In this context in particular, sampling biases due to self-selection or convenient sampling of participants [54], the trustworthiness of data, as well as the quality of data analysis represent critical points. Interestingly, even the strictest advocates of participatory research do not require patients or other lay people to take on the lead in research, but rather envision an ongoing cooperation between researchers and participants throughout the research process [18, 36]. Consequently, if some initiatives in HBDR assign patients a leading role, this may exceed the ethically desirable dimension of *par*-ticipation and *co*-determination in the sense of a symmetrical collaborative process. In particular, in light of the aforementioned challenges, there is need for adapting the standards of ethical oversight to the context of participant-led research [17].

An illustrative example of participants’ role as co-decision-makers is the recent Swiss initiative *MIDATA*. Organized as a non-commercial data cooperative, it provides an innovative IT infrastructure for overcoming the limitations of individual control over data. In *MIDATA*, participants own their data as a collective, collectively determine the conditions for data use by others (e.g., researchers) and have a say on how the revenues of data use by third parties will be reinvested in future research endeavours [55, 56]. People are thus not only provided with an exit option from research they do not agree with; they can also determine the conditions of data use in a bottom-up approach. As an innovative approach of involvement, such data cooperatives have been described as a grand experiment in democratic data self-governance [57]. It is important to note, however, that participants of the *MIDATA* platform are not necessarily involved in the research process as such. As the description of a current research project involving patients after bariatric surgery reveals, the role of patients is to provide data in the first place. However, in contrast to *Patients.Like.Me*, they, as a collective, have the power to determine the framework of using these data in research [58].

Even though such data cooperatives can be seen as a promising way to realize co-decision-making regarding research, they place high demands on participants. Specifically, active participation in data cooperatives requires abilities (social, educational) and resources (e.g., time) that may prevent certain groups from participation and lead to an overrepresentation of others. Therefore, in order to avoid that the conditions of research will only be determined by those with the most favourable social, economic and educational prerequisites, such barriers would have to be removed [56]. The promotion of digital health literacy in the broader public may be one step to achieve this [59].

### Conclusions: taking participation seriously - ethical points to consider in future HBDR and other data-intensive medical research

While participatory promises are booming in the context of HBDR and other kinds of data-intensive research in medicine and healthcare, our analysis reveals specific deviations from the original normative spirit of participation that create ethical challenges and practical problems. In this field of research, transparency about the use of data is often framed as a participatory practice in and of itself. Of course, the promotion of an open and transparent research culture is an important concern [60, 61]. From a political-ethical perspective, however, the provision of information only qualifies as a minimum prerequisite for more far-reaching and substantial forms of patient participation.

Moreover, we observe a certain tendency to replace crucial conditions of participation in politics and bioethics, such as collective deliberation and consultation, with individual decision-making via online-tools. From an ethical perspective, this use of online-tools hardly differs from conventional forms of research participation and disregards the normative claims of real participatory efforts. At the same time, albeit much less frequently, data-intensive research can also evoke overambitious and ethically problematic participatory notions, for example, by highlighting research trials led by patients as innovative science. Both possibilities represent extremes that need to be avoided. On one hand, participation should not be reduced to a toothless paper tiger simply asserting obvious legal standards of consent. On the other, however, it is also problematic to evoke highly ambitious participatory promises that are difficult to keep due to ethical, practical and methodological limits of citizen science in this research context. Therefore, in light of the original normative implications of participation, future usages of the concept should be more thoughtful and appropriate. Specifically, the following five points are worth considering and may also be relevant for participation and PPI in medical research in general:

First, data-intensive research projects should critically reflect the underlying motivation for appealing to participatory notions. In particular, it is necessary to distinguish between merely instrumental uses, for example in order to increase the volume of data or acquire financial gains, and genuinely normative reason for involving patients in order to adopt research aims to patients’ opinions. Raising awareness regarding this distinction has immediate practical implications for the role patients adopt in this context. Specifically, if participatory notions are primarily invoked to motivate data donation and to legitimize research, participants remain largely passive subjects [38]. Such “token public involvement” [62] may generate significant mistrust if the public notes failures in achieving this goal [7]. Moreover, it is important to critically reflect on the ethical need of patients or subjects becoming involved as co-researchers. Given the challenges of data privacy or potential misuse in data-intensive research, much would be gained if patients were involved in the *governance* of such research initiatives (e.g., as members of a project advisory board, or regularly invited to workshops).

Secondly, as data-intensive research initiatives build on the use of data that patients provide not least with the intent to improve their own health, it is important to become more transparent about whether patients participate for their own medical benefit or whether participatory notions are evoked in the sense of public engagement that goes beyond individual purposes [63]. Admittedly, HBDR and other kinds of data-intensive research in medicine and healthcare are increasingly blurring the boundaries of clinical care and research. Consequently, it is not only important to avoid therapeutic misconceptions (a well-known problem in other research contexts [64]), but also ‘participatory misconceptions’. Such misconceptions occur if research initiatives use public engagement rhetoric without actually empowering patients as genuine partners in research. In fact, although it may be beneficial to individual patients to receive information on their individual health status, it is misleading to frame this in terms of patient involvement in research.

Therefore, as a third requirement, research initiatives should resist the tendency to depoliticize the notion of participation by denying patients real influence on research – or even exploiting their engagement in order to outsource research work and save time and money. As Prainsack argues, “the words that we use to describe such practices should be sensitive to the power structures that they are embedded in. We should speak of responsibilities devolved to patients, invited participation, and again, of ‘patient work’” [19]. However, while Prainsack criticizes that participation is often invoked in instances where people do what public or other collective actors should be doing [19], in the context of data-intensive medical research, this is only half the story. Specifically, there may be nothing wrong with patient-reported data as such, provided patients receive sufficient information. Ethical problems may arise, however, if patients are led to believe (e.g., by sophisticated online platforms) that they can take their medical destinies into their own hands, while it is actually others (researchers, commercial agencies) who are in charge. Taken to its logical end, this use of participatory rhetoric may even decrease current research ethical standards, as it only pretends that individuals hold decisional powers.

A fourth point to consider relates to the availability of resources necessary for realizing genuine participation. For example, if patients are expected to participate in the governance of data-intensive research, research projects should be prepared to hold sufficient financial and structural resources to allow patients, lay persons or patient representatives to effectively communicate and express their opinions. Moreover, the provision of resources should be planned accordingly and be balanced in order to avoid conflicts of interests and compromises to patients’ credibility. In addition, given that researchers are usually paid or reimbursed for their work, an appropriate acknowledgement and even reimbursement of patients’ contributions as citizen researchers should be taken into consideration as well. Ultimately, equal access to funding and relevant sources of knowledge production would be a necessary precondition for true power balance and equal status.

Finally, a general concern that may remain is that even the most sophisticated participatory approaches have a certain affirmative tendency, i.e., an inclination to presuppose and reinforce basic agreement with and support for new technological developments rather than objection or rejection. Hence, on a collective level, the public expression of basic concerns about, or even disagreement with, such technological developments may only be possible outside of the respective initiatives. If the political discourse does not allow for an inclusion of such sceptical and dissenting voices, this might result in silent resistance, fundamental opposition or even active boycott. In order to realize participation in data-intensive medical research such as HBDR in an authentic, adequate and coherent way, systematic consideration of its ethical, legal and social implications, e.g., by the inclusion of ELSI projects in big data research initiatives, would be an important step towards making future research in this field ethically, legally and socially robust.

## Take home messages

- References to patient or citizen participation and involvement in HBDR and other data-intensive research are often vague, merely rhetorical, or oblivious to normative implications.
- To ensure the integrity of research, it is necessary to distinguish between merely strategic uses of participatory rhetoric, for example, to increase data volume or financial benefits, and genuinely normative reasons for involving patients.
- Research initiatives need to be transparent about the aim, extent and benefits of participation in order to avoid ‘participatory misconception’ resulting in misleading expectations of participants regarding their power.
- Inflationary use of participatory rhetoric can undermine public trust in data-intensive research initiatives, especially if participation eventually boils down to simple forms of consent for or denial of data collection, access and use.
- Financial resources, as well as education, time and communicative skills for deliberation should be considered within research plans and budgets to enable real cooperation and involvement of patients or citizens.

## Abbreviations

ALS: Amyotrophic Lateral Sclerosis
ELSI: Ethical, Legal, Social Issues
HBDR: Health-related Big Data Research
NHS: National Health Service
PPI: Patient and Public Involvement

## Footnotes

1 While participation is often primarily used regarding patients, healthy subjects are of equal importance in HBDR and data-intensive medical research. Therefore, speaking of patients in the following includes healthy subjects and vice versa.

2 The role of a co-researcher may involve further differentiations. In particular, regarding the extent of involvement, one could distinguish between patients deciding over the direction of research and patients performing research tasks themselves.

3 Notably, there are external driving forces behind this development, insofar as current research funding programs (e.g., the EU commission) increasingly require the involvement of patients and the public in general.

4 This is in line with what Chung and Lounsburry describe as “empowering co-investigation” [31].

5 In addition, the app automatically tracks the user’s location in order to match his health data with air-quality reports for the specific region.

6 In their analysis of patient-centered initiatives, Kaye and colleagues also classify TuAnalyze as an instance of “Citizen Science” [14]. This might be due to the fact that the network itself has been initiated by a patient. However, regarding the role of patients who participate in this network, it seems closer to what they describe as “matchmaking” between patients and researchers.
